# Restoring the missing person to personalized medicine and precision psychiatry

**DOI:** 10.3389/fnins.2023.1041433

**Published:** 2023-02-09

**Authors:** Ana Gómez-Carrillo, Vincent Paquin, Guillaume Dumas, Laurence J. Kirmayer

**Affiliations:** ^1^Culture, Mind, and Brain Program, Division of Social and Transcultural Psychiatry, Department of Psychiatry, McGill University, Montreal, QC, Canada; ^2^Culture and Mental Health Research Unit, Lady Davis Institute, Jewish General Hospital, Montreal, QC, Canada; ^3^Precision Psychiatry and Social Physiology Laboratory at the CHU Sainte-Justine Research Center, Université de Montréal, Montreal, QC, Canada; ^4^Mila–Quebec Artificial Intelligence Institute, Montreal, QC, Canada

**Keywords:** precision psychiatry, personalized medicine, person-centered psychiatry, multilevel explanation, ecosocial approach

## Abstract

Precision psychiatry has emerged as part of the shift to personalized medicine and builds on frameworks such as the U.S. National Institute of Mental Health Research Domain Criteria (RDoC), multilevel biological “omics” data and, most recently, computational psychiatry. The shift is prompted by the realization that a one-size-fits all approach is inadequate to guide clinical care because people differ in ways that are not captured by broad diagnostic categories. One of the first steps in developing this personalized approach to treatment was the use of genetic markers to guide pharmacotherapeutics based on predictions of pharmacological response or non-response, and the potential risk of adverse drug reactions. Advances in technology have made a greater degree of specificity or precision potentially more attainable. To date, however, the search for precision has largely focused on biological parameters. Psychiatric disorders involve multi-level dynamics that require measures of phenomenological, psychological, behavioral, social structural, and cultural dimensions. This points to the need to develop more fine-grained analyses of experience, self-construal, illness narratives, interpersonal interactional dynamics, and social contexts and determinants of health. In this paper, we review the limitations of precision psychiatry arguing that it cannot reach its goal if it does not include core elements of the processes that give rise to psychopathological states, which include the agency and experience of the person. Drawing from contemporary systems biology, social epidemiology, developmental psychology, and cognitive science, we propose a cultural-ecosocial approach to integrating precision psychiatry with person-centered care.

## Introduction

Recently, much interest has centered on efforts to develop a more personalized or precision psychiatry by employing genomics, brain imaging, and other technologies to identify biomarkers that can guide differential diagnosis and tailor patient-centered therapeutics (Friston et al., 2017; Bzdok et al., 2021). With few exceptions, however, these advances have not been widely implemented and have had little impact on psychiatric clinical practice. This limited uptake may reflect their recency, the need for further replication, and the logistical complexities and cost of implementation (Baldwin et al., 2022). But, as we will argue in this paper, there are reasons to think these methods alone will not be sufficient to enable a robust, precise, and person-centered psychiatry.

Although personalized and precision psychiatry holds promise, it faces practical and conceptual challenges. With the current state of knowledge, the biological markers that are intended to yield more precise assessments often remain at the correlational level and do not reveal causal pathways that can be modified by treatment interventions. Moreover, the statistical significance of those biomarkers does not necessarily translate into clinical significance (Loth et al., 2021). Finally, the predictive utility of measures used to characterize patients and their disorders is inherently limited because they do not capture crucial dimensions of patients’ illness experience, self-understanding, social contexts, personal priorities, or concerns.

While “omics” and brain imaging are being harnessed to develop precision psychiatry, if real precision is to be achieved, diagnostic assessment and treatment will need to go beyond neural signatures or biotypes, to be person-centered and context-sensitive. In this paper, we outline how a person-centered, cultural-ecosocial perspective (Kirmayer, 2019) can address some of the limitations of current personalized or precision approaches to psychiatric research and practice.

## The logic of personalized medicine and precision psychiatry

Personalized medicine and precision psychiatry aim to refine diagnostic assessment and interventions to make them more accurately reflect and respond to the health status of individuals (Fernandes et al., 2017; Williams and Hack, 2021). The goal is not so much tailoring interventions to individuals as improving assessment of prognosis and treatment selection through better characterization of aspects of biology relevant to mental disorders. The logic of this approach starts with the recognition that biology is a source not just of universal features of human physiology but also of individual variation. The new technologies of neuroimaging and multi-omics (i.e., genomics, transcriptomics, proteomics, metabolomics, etc.) allow us to characterize individuals at multiple levels with high-dimensional data (e.g., functional MRI and genome-wide association studies; GWAS) collected from very large samples. The massive datasets that result pose challenges for data management and analysis, but advances in computing, artificial intelligence, and machine learning have provided methods to translate them into biomarkers. If these biomarkers can be correlated with differential disease course, treatment response and outcome, we can develop pragmatic typologies and more targeted treatment interventions.

Unfortunately, the search for biomarkers for the disorders identified in current psychiatric nosologies like the Diagnostic and Statistical Manual (DSM) of the American Psychiatric Association and the World Health Organization International Classification of Diseases (ICD) has had limited success to date. While many correlates have been found, few have the level of sensitivity or specificity that would make them useful as diagnostic indicators, measures of clinical course, or guides to treatment selection (García-Gutiérrez et al., 2020; Loth et al., 2021). This failure has been attributed to the heterogeneity of clinical syndromes and has led to several strategies to rethink nosology in ways that would reflect more homogeneous categories of mental disorders at the biological level. Newer research approaches thus aim to identify biological traits (endophenotypes) or mechanisms that underlie phenotypic expressions of psychopathology, thereby defining new categories of disorders either within or across existing diagnostic entities (Gottesman and Gould, 2003; Kendler and Neale, 2010; Kotov et al., 2021; Maes, 2022).

The development of the Research Domain Criteria (RDoC) by the U.S. National Institute of Mental Health was a major effort to re-orient research by prioritizing studies that begin with an identified biobehavioral system (often one that can be studied in animal models) rather than a discrete clinically identified disorder (Insel et al., 2010). The RDoC scheme was originally presented as a 2D grid in which the rows represent a series of “domains” or specific biobehavioral systems (e.g., sensorimotor, cognitive, arousal/regulatory, etc.), and the columns identify the potential units or levels of analysis from molecular biology, through cellular, neuronal circuitry, and brain network characteristics, to self-reports of experience. Each of these dimensions can be expanded by extending their scale to include social processes and social levels of analysis, as well as by adding a third temporal dimension of lifespan development, which would include early neurodevelopmental, learning, and social processes, as well as cultural, social, and historical processes that shape individual experience across the lifespan. RDoC has reserved a place for these larger scale processes beyond the individual as a separate dimension, labeled “environment”^1^ and, although it remains to be elaborated, recent efforts to unpack this dimension identify multiple parameters related to illness course, treatment response and outcome (e.g., Carter et al., 2021). The importance of the social environment for the onset, course, and outcome of psychiatric disorders is evident from a wealth of research on social determinants of mental health (Alegría et al., 2018; Jeste and Pender, 2022; Sommer and DeLisi, 2022). However, incorporating this dimension in research requires careful elaboration and consideration of context that goes beyond a simple checklist of environmental factors or indicators. A recent study by Ku et al. (2022) illustrates the importance of social and cultural processes in moderating the impact of social determinants on neurobiological measures. Using data from a longitudinal study in North America, Ku et al. (2022) found that higher levels of neighborhood poverty were associated with reduced hippocampal volume in individuals at clinical high risk for psychosis, but this relationship was significantly moderated by social engagement: higher levels of social engagement were protective against hippocampal volume reduction.

Precision psychiatry builds on theoretical research frameworks like the RDoC to identify measures of pathophysiological mechanisms that can be targeted with specific interventions (Fernandes et al., 2017). The clinical assessment of biotypes and processes associated with particular neural circuits has the potential to shift psychiatric practice away from treatment based on heterogeneous categories toward more precisely matched interventions (Beam et al., 2021). Bringing together measures from diverse scientific approaches, including multiomics, neuroimaging, cognition, and clinical characteristics using systems biology and computational psychiatry tools is one step toward precision in diagnostic classification and treatment selection.

While RDoC aims to eventually develop a new nosology, other researchers continue to look for biomarkers that can refine the use of existing nosologies in the DSM or ICD by identifying subtypes, prognostic or therapeutic response indicators. *Pragmatic neuroscience* is an approach to precision psychiatry that aims to develop “neuroscience-based objective quantitative markers that aid clinical decision-making and have implications for individual patients, for example by objectifying diagnosis, quantifying prognosis, supporting treatment selection, and yielding objective severity markers for disease monitoring” (Steele and Paulus, 2019, p. 404). The hope is that this can be achieved without having to refine psychiatric nosology or clarify illness mechanisms because it can be based on identifying “objective” (i.e., reliable) covariates (Steele and Paulus, 2019, p. 405). Potential advances in clinical translation of neuroscience include using neuroimaging measures in combination with machine learning (Phillips, 2012; Etkin, 2019; Nielsen et al., 2020) to facilitate differential diagnosis between unipolar and bipolar depression (Han et al., 2019: Colombo et al., 2022), estimate illness prognosis (Janssen et al., 2018), and predict treatment response (Phillips et al., 2015). Unfortunately, the pragmatic approach to prognosis has had limited success to date, but researchers remain optimistic (Zeier et al., 2018; Rose et al., 2022).

A largely unquestioned assumption within precision psychiatry is that mental disorders are essentially brain disorders and can be adequately characterized in terms of “objective” brain dysfunction (Insel and Cuthbert, 2015). This assumption underwrites the confidence expressed by many advocates for precision psychiatry that it holds the keys to a vastly more effective clinical approach. As Salagre and Vieta (2021) put it: “Precision psychiatry will eventually deliver because there is no question, in our opinion, that mental disorders are disorders of the brain, and as such, can be tracked through biological clues, which can be complex, but are still there, awaiting discovery” (p. 1413). As we shall argue, however, there are many reasons to think that processes beyond the brain can constitute, cause, shape, and resolve psychiatric disorders (Borsboom et al., 2019; Köhne and van Os, 2021). Without systemic consideration of qualitative, subjective, intersubjective, and contextual dimensions, the predictive value of “objective” covariates or biomarkers will be limited. There is an urgent need, therefore, for an ontology that includes these larger processes to guide psychiatric research and practice (Kirmayer and Gold, 2012; Kirmayer and Crafa, 2014; Paris and Kirmayer, 2016; van Os and Kohne, 2021).

## The ambit of precision psychiatry and the need for multiple approaches

Psychiatric disorders are not a single type of entity. Current nosology groups together very different types of health conditions, including symptoms, syndromes, diseases, and “problems in living” or maladaptation. Clearly, precision psychiatry cannot be applied in the precisely same way to different types of psychiatric disorders, but there are broad claims for its utility in the literature—as there are for RDoC. Advocates for precision psychiatry routinely argue that the approach can be applied to common mental disorders including depression, anxiety and stress-related disorders (e.g., Fernandes et al., 2017; Williams and Hack, 2021). For example, a recent article suggests that “polygenic risk scores may be useful for prediction of vulnerability to depression and resilience under stress” (Kambeitz-Ilankovic et al., 2022).

Different types of psychiatric entities have prompted different modes of explanation. McHugh and Slavney (1983/1998) argued that psychiatry must employ multiple perspectives to make sense of patients suffering and respond effectively. They argued for the complementarity of four perspectives: *disease* (brain dysfunction or disorders), *dimension* (e.g., personality traits), *behavior* (i.e., motivation or goal-orientation), and *life story* (biographical events and trajectories characterized in terms of setting, sequence and outcome). The “perspectives approach” could be misconstrued to mean that there are different types of psychiatric problems that warrant entirely different types of explanation with corresponding types of treatment—reflecting an ontological distinction that is underwritten by mind-body dualism (Slavney and McHugh, 1987/2016). While this dualism persists in psychiatric thinking as well as everyday explanation (Kirmayer, 1988; Miresco and Kirmayer, 2006), it does not reflect the ways in which brain function, individual traits, goals, life narratives and predicaments interact to give rise to particular problems. The complex causality and many gradations of agency seen in clinical cases underscore the need to integrate these perspectives (Kirmayer and Gómez-Carrillo, 2019).

Some disorders, like autism or schizophrenia, are commonly assumed to have a stronger neurobiological basis relative to other disorders like personality or adjustment disorders—and there has been some progress in identifying biological correlates with potential clinical relevance. For example, rare coding genetic variants have been shown to have a substantial effect on the risk for schizophrenia (Singh et al., 2022), and a transdiagnostic stratification of patients with recent-onset depression or psychosis was found to support the clinical relevance of neurobiological phenotypes (Lalousis et al., 2022). However, while there may be rare genetic variants that confer high risk, most of the susceptibility genes identified to date contribute only very small amounts of elevated risk to many different disorders and may act *via* final common pathways that are also influenced by other non-genetic factors (Smoller et al., 2019; Rees et al., 2021; Liu and Lau, 2022).

In the case of autism, the nosological category includes a broad range of conditions that may have different causes and a wide range of clinical manifestations and trajectories (Eyal, 2010; Lombardo et al., 2019; Mottron and Bzdok, 2020). For example, there are rare syndromic forms like Phelan-McDermid that are closely related to a specific genetic variant and these may eventually benefit from targeted pharmacological intervention (Dyar et al., 2021), while other highly heterogeneous forms of autism may be better approached with environmental interventions. However, for the same condition, the impact of a genetic variation or other biological factor may change across lifespan through epigenetic modifications in response to environmental contexts (Assary et al., 2018; Richetto and Meyer, 2021). Moreover, the impact of genetic variation may be modulated by other factors such as gender and culture (Lai et al., 2015; Kwon and Sasaki, 2019; McQuaid et al., 2019).

In fact, the distinction between biological or genetic on the one hand and psychological on the other does not hold up to close scrutiny (Bienvenu et al., 2011). Biological, psychological and social processes are thoroughly intertwined in human experience and in any specific instance of psychiatric disorder or dysfunction. Neither adaptive functioning nor broad heterogenous categories of mental disorders can be described as more or less biological. At a population level, any apportioning of degree of biological, psychological or social importance can only be relative to specific instances, where some factors are fixed while others vary. At an individual level, such judgments are made for pragmatic reasons including sociomoral meaning and available interventions (Kirmayer and Gómez-Carrillo, 2019). Hence, the multiple perspectives identified by McHugh and Slavney (1983/1998) are best seen as addressing different facets of problems that need to be integrated to provide more “precise” multilevel, multimodal care. The challenge for psychiatry, then, is to understand these perspectives not simply as complementary but as pointing to facets of the same dynamical system.

Individuals’ self-understanding, framed in terms of causal attributions, metaphors and more extended illness narratives, influence symptom interpretation, help seeking and treatment response (Kirmayer et al., 2023). Diagnostic labels and medical explanations themselves become part of illness narratives and identities that shape illness experience, coping and adaptation. This breaks down Jaspers’s (1913/1997) distinction between meaning and explanation in psychiatry. The stories that patients and clinicians generate influence behavior and adaptation as well as the social response of others in ways that determine illness outcomes. Psychiatric understanding then requires both mechanistic and narrative modes of explanation, which are linked through meaning and sense-making processes (Kirmayer, 2015a). These processes, in turn, depend on cultural concepts of the person.

## Missing persons: The limits of precision psychiatry

Concepts of the person are social constructions that vary across cultures and depend crucially on social context and interactions with others (Kirmayer, 2007; Kirmayer et al., 2018). Our everyday notions of personhood center on experiences of subjectivity, agency, social relationships, life plans or projects, and moral accountability articulated through narrative practices. These dimensions of personhood give rise to a sense of self, identity, and personal history or autobiography, which we actively use in making sense of illness experience, coping, and adaptation. Unfortunately, none of this is well-represented in current approaches to precision psychiatry. The notion of person implicit in precision psychiatry and personalized medicine reduces the person to an isolated individual organism, disembedded from social context, who can be adequately characterized by variation across multiple biological dimensions or parameters. Given the powerful effects of social context, experience, and self-reflexivity in psychopathology, this stripped-down version of the person poses important limitations on what precision psychiatry can achieve.

In precision psychiatry, the characterization of individuals is currently done mainly using biological parameters similar to those employed in other areas of medicine. Four broad sets of critiques suggest limits to what this approach can accomplish: (i) the difficulty of identifying biomarkers for multilevel disorders; (ii) the potential limitations in finding underlying causal factors because of the supervening dynamics of symptom networks; (iii) the lack of attention to developmental trajectories and social-environmental contexts; and (iv) the effects of agency and subjectivity on illness experience, course, and outcome.

As Tabb and Lemoine (2021) point out, precision psychiatry is modeled on the success of precision medicine in oncology and cardiology. In those medical specialties, specific biomarkers have been identified that are closely associated with the underlying mechanisms of pathophysiology and, hence, can not only guide treatment choice and predict the response to specific interventions but also contribute to the development of novel and, in some cases, personalized treatments. In contrast, the search for biomarkers in psychiatry generally has yielded evidence of many biological factors, each of which has only small correlations with the symptoms, course, or outcome of many different disorders. At present, most of these putative biomarkers lack adequate diagnostic and predictive accuracy and are not clearly linked to specific mechanisms of pathophysiology or psychopathology.

This difficulty in finding biomarkers that are diagnostically useful has been blamed on using clinically derived diagnostic categories that are heterogeneous and reflect behavioral phenotypes that are far from potential underlying neurobiological mechanisms. The lack of specific biomarkers may not be simply because the syndromes or diagnostic constructs are ill-defined or based on variable phenomenology, but because the causal mechanisms of disorders result from interactions in networks of symptoms that have complex dynamics unfolding over time (van Os et al., 2013; Borsboom et al., 2019; Bringmann et al., 2022). If so, there may be no single process explaining all the features of the disorder; rather, the disorder emerges from interactive processes located at different levels, from the biological to the social (Borsboom, 2022).

Of course, there may be some disorders for which a dominant cause (genetic, structural, or functional) will be identified, but even in these cases, contextual factors remain crucial. A classic example is phenylketonuria (PKU), in which mutations in the phenylalanine hydroxylase gene only cause neuronal injury in the presence of dietary phenylalanine in the environment—both genetic and environmental factors need to be present for the disorder to manifest (Widaman, 2009). Hence, even a disorder with Mendelian inheritance can have complex interactions with other genes and with the environment. Given the fact that most psychiatric disorders appear to have many genetic factors that each make a small contribution to the phenotype (Sullivan and Geschwind, 2019; Rees and Owen, 2020), the influence of any one factor is even more likely to depend crucially on its context within the whole genome, its epigenetic regulation, and the phenotype (“the body”), as well as the physical and social environment. This likelihood that most psychiatric disorders (even those with evidence for high heritability) involve complex person-environment interactions makes it unlikely that research will be able to find the same close link between pathobiology and clinical syndromes that has occurred for some cancers and other conditions.

A second and related critique stems from the realization that symptoms (experiences and behaviors) may arise from multiple mechanisms and may interact in ways that lead to mutual amplification or exacerbation. In effect, mental disorders may not be discrete conditions with single underlying mechanisms but may result from interactions within symptom networks that have their own dynamic properties (Borsboom et al., 2019). These networks may involve not only interactions among multiple neurobiological subsystems, but also cognitive and interpersonal interactions that arise because of what symptoms mean to individuals and to others around them. For example, in a putative network model of depression, the feeling of worthlessness causes suicidal ideation, which may further exacerbate the feeling of worthlessness; each of the two symptoms and their mutual interaction is multiply determined by biological factors (e.g., genetic makeup, brain circuits), psychological factors (e.g., coping strategies), and social contextual (e.g., recent exposure to suicide) and cultural factors (e.g., the meaning and acceptability of suicide) (Borsboom et al., 2019). Conscious experience itself can participate in network dynamics (Liu and Lau, 2022). Moreover, symptoms also may function as behaviors or communications in interpersonal interactions, adding layers of social dynamics (Kirmayer, 2022). This network perspective challenges the assumption that mental disorders are simply brain disorders and points to the need for a broader systemic view.

Social and cultural factors can also influence how biomarkers map onto mental disorders or symptoms. Statistical associations between biomarkers and psychiatric outcomes may be confounded or moderated by environmental contingencies. For example, geographic region is a potential source of confounding in GWAS, likely because of socio-economic stratification between regions; these regional differences are correlated with polygenic signals, thereby inflating heritability estimates of complex behavioral traits (Abdellaoui et al., 2022). There is evidence that the historical period of birth moderates environmental and genetic influences on complex phenotypes, possibly as a result of changing social contexts (Min et al., 2013; Tucker-Drob et al., 2013; Silventoinen et al., 2020). For example, in a twin study, the heritability of educational attainment was greater among individuals born in 1900–1949 than among individuals born in 1950–1989, with a corresponding smaller influence of shared environments (Silventoinen et al., 2020). Social reforms, economic growth, and other environmental upheavals have been invoked to explain interactions of heritability with the period of birth (Min et al., 2013; Tucker-Drob et al., 2013).

The third critique concerns the lack of attention to developmental trajectories and contexts (Garber and Bradshaw, 2020; Conradt et al., 2021; Hitchcock et al., 2022). The architecture of the brain results from neurodevelopmental processes involving ongoing interactions with the immediate environment at each step or stage (Hiesinger, 2021). These developmental influences of the environment on the brain are redoubled by the multiple forms of memory and learning and allow the brain to adapt to changing contingencies. The functional anatomy of the brain is thus not simply a result of its phylogenetically determined organization but reflects each individual’s distinctive developmental and learning history. This means that, insofar as they represent the outcome of developmental events and learning, mental health problems do not just reflect alterations in brain circuitry but involve developmental interactions that can only be captured by multidimensional causal models that include environmental factors.

Although, as we note above, the RDoC and other recent frameworks for precision psychiatry acknowledge the importance of environmental factors, they have not developed frameworks that systematically assay the environment or that characterize environment-person interactions (Cuthbert, 2022). Efforts to include personal or social-environmental dimensions have been limited in precision psychiatry. In response, a new line of epidemiological research attempts to aggregate environmental exposures and consider their joint associations with mental health and illness across clinical and non-clinical samples. For example, constructs like the “exposome” or “envirome” consider the simultaneous contributions of household and socioeconomic adversity, neighborhood environment, day-to-day experiences, family values, perinatal complications, and other exposures to mental health problems (Guloksuz et al., 2018; Hullam et al., 2019; Liu et al., 2022; Pries et al., 2022). The notion of “exposome” was introduced originally in relation to environmental toxicology and was then extended to adverse events and other environmental factors (Wild, 2012; Vermeulen et al., 2020; Vineis et al., 2020). The concept of exposure encourages us to read the environment from the point of view of its impact on the individual (setting aside the profound ways in which humans construct their own global and local ecological niches, for better or for worse). But the social environment has its own structure and dynamics that may differentially yield stressors and resources for different individuals or for whole groups that occupy particular social positions. While the envirome can be expanded to include a wide range of discrete factors identified on the basis of epidemiological or clinical studies, in reality, these factors are not independent but interact with each other and with the person’s experiences and behaviors in complex ways that reflect the local dynamics of social structure. The concept of *syndemics* aims to foreground these deep connections between co-occurring forms of social disadvantage and illness (Singer et al., 2017), building on work in social epidemiology (Krieger, 2021), and public health (El-Sayed and Galea, 2017). These interactions complicate recent calls for the development of a “polysocial” risk score (Figueroa et al., 2020). The predictive value of such a score will vary with local social contexts, which influence the ways in which factors interact.

As discussed above, symptoms of psychopathology may constitute networks or systems with their own dynamics, giving rise to some of the syndromes characterized in psychiatric nosology. Social factors are key to these interactions. Social structural issues, including poverty, racism and discrimination, marginalized identity, and urban living all correlate with mental health problems more strongly than do biological factors (Wallack and Thornburg, 2016; Glasgow et al., 2018; Anglin et al., 2021; Insel, 2022). Measures of single levels or dimensions— whether these be structural differences in regional brain volumes or patterns of activation found on brain imaging, genetic markers, experimental task outcomes, or psychometric scales—will therefore have limited ability to capture the process of symptom production, illness experience, and treatment response.

Finally, current multifactorial models of psychopathology often treat the person as the passive locus on which diverse factors converge to affect the brain. Missing from these schemes is systematic attention to the individual as a person, with subjectivity, self-reflection, and agency that must be captured through attention to phenomenology, narratives, personal history, values, and other aspects of identity and experience in context (Haslam et al., 2021). For example, the highest level of organization explicitly included in RDoC is “self-report”—which reflects the articulation of experience through language mainly in terms of specific questionnaires and quantitative measures. There is no explicit place for or specification of the rich narrative and metaphoric modes of illness experience that may be central to how individuals make sense of their experience and convey it to others. Crucially, this articulation of experience is usually intersubjective, depending on the interpersonal context of communication that influences self-understanding, self-construal, social presentation, and positioning (Dumas et al., 2020; Dumas, 2022). In the RDoC scheme, both the rich phenomenology of experience and its socio-cultural embedding are nowhere to be found—nor is there any place for the many existential and social predicaments people face that are important causal factors of illness, mediators of pathology, and objects of clinical attention in their own right.

## Challenges to precision in psychiatric research and practice

There are multiple methodological and epistemic challenges to achieving precision in psychiatry that represent not simply practical obstacles or the limitations of current approaches but reflect the central role of meaning and experience in psychopathology (Berrios and Marková, 2015) and, hence, point directly to the value of a more context-sensitive, integrative, idiographic approach.

### Reproducibility and generalizability

The reproducibility of research findings in precision psychiatry has been limited (Bzdok and Ioannidis, 2019). This may reflect several related issues: (i) heterogeneity in research and clinical populations; (ii) failure to control for important sources of variance; and (iii) sensitivity of relationships to contextual factors. The sample sizes in neuroimaging studies tend to be small and not representative of the diversity of clinical populations (Phillips, 2012; Falk et al., 2013). Creating large datasets can address this limitation, but the data collected needs to be representative of the populations to which findings will be applied and include relevant markers of social and cultural identity or situation to guide translation. Currently, most biological research in psychiatric research involves “WEIRD” (Western, educated, industrialized, rich, and democratic) samples that are not representative of the global population nor of the racialized and minoritized groups (Henrich et al., 2010). The latter groups may be at higher risk for specific forms of adversity (e.g., discrimination), associated mental health problems, and inequities in health services, including limited access, misdiagnosis, and inadequate treatment (Nazroo et al., 2020). Failure to capture these dimensions can lead to misinterpretation of findings and misattribution of differences to group characteristics rather than contextual variables.

### Ecological validity

Translating knowledge gained from research at the population level or from experimental samples to the clinical assessment and treatment of individuals requires considering the right parameters. Laboratory tasks and experimental research often deploy highly controlled conditions to isolate the associations of interests, such as the activation of a specific brain region in response to an intervention or according to illness status (Nastase et al., 2020). The limitation of this approach is that it does not account for the diversity of environmental conditions in the real world, which can modify or mediate some of the associations that are being studied. For example, a range of observational and experimental approaches have shown that social connections influence mental health problems across the life span–not only relative to their risk of onset, but also their subsequent course and biological correlates (Bzdok and Dunbar, 2020, 2022). Including such social determinants in multiomics models would allow controlling for variation that might otherwise be misinterpreted as measurement error, random variation or “noise” and improve the detection of causal relationships.

Moreover, precision psychiatry has inherited biases from computational psychiatry, such as its over-emphasis on decision-making using idealized tasks derived from economics and game theory (Montague et al., 2012; Series, 2020). As a result, biological markers or interventions identified in laboratory experimental settings may not generalize well to naturalistic conditions. Ecological validity can be improved by using research designs that better capture these real-world contexts (Fan et al., 2021). The research community has much to gain by integrating feedback from stakeholders to design experiments that capture essential elements of the ecological niche of patients and families and that address their priorities and concerns (Filipe et al., 2021; Gauld et al., 2022). Even when this is not possible, adequately characterizing the ecological niche of study participants, and examining the moderating effects of contextual variables, will allow researchers to uncover context-specific effects (Holleman et al., 2020).

Ecological momentary assessments and digital phenotyping can be used to enhance ecological validity in psychopathology research (Robinaugh et al., 2020; Carmi et al., 2022; Verhagen et al., 2022). Ecological momentary assessments are brief questionnaires that are administered repeatedly at close intervals (e.g., multiple times daily) to investigate how unfolding experiences, behaviors, and environmental exposures influence each other over time (Myin-Germeys et al., 2018). Ecological validity is preserved because the questionnaires are administered remotely, typically *via* the participants’ smartphone. These subjective reports can be complemented with digital phenotyping, which consists of “objective” data collected from mobile sensors that is used to infer physiological, mental, or environmental states (Mohr et al., 2017). Examples of mobile sensing include accelerometry, geolocation, and phone calls to elicit information about the immediate environment. In ecological momentary and digital phenotyping research, repeated assessments can be analyzed at the within-person level, where individuals are their own comparators over time. Under appropriate conditions, this approach can provide a stronger basis for causal inference than between-person analyses of observational studies.

Ecological validity also depends on characterizing the social context of individuals adequately and incorporating this data into multilevel analysis. Diverse statistical methods are available to do this, including multilevel factor analysis, multilevel structural equation modeling, and dynamical systems approaches (Dunn et al., 2014; Barker et al., 2020). These methods can be used to identify key social determinants of health and the impact of environmental factors at family, neighborhood, region, society or transnational network levels—each of which may reveal mechanisms of pathology and potential sites for population health intervention as well as individual treatment (El-Sayed and Galea, 2017; Reuben et al., 2020).

### Inference from group differences to individual cases

Clinical practice involves a process of translating scientific knowledge identified in studies of experimental groups or populations into specific approaches for an individual. This can be framed as a move from general (*nomothetic*) knowledge of processes to particular (*idiographic*) explanation in the formulation of an individual case. Group-level statistical significance is not the same as individual patient clinical significance. The problem of misinterpreting population-level correlations as evidence of individual causal links is termed “the ecological fallacy” and is widespread in neuroscience research (Cragg et al., 2019). Even when potential links are identified, differences in markers measured at the group level often do not have sufficient predictive accuracy to translate into clinically useful information at the individual patient management level (Steele and Paulus, 2019; Loth et al., 2021). Statistical methods that work for identifying putative mechanisms may not be useful for clinical prediction (Bzdok and Ioannidis, 2019).

There is emerging idiographic research in psychiatry that can enhance our capacity to tailor mental health care to the individual. Examples of these approaches include N-of-1 trials, idiographic network analyses, and idiographic digital phenotyping. N-of-1 trials are experimental studies conducted in a single individual, with different interventions tested sequentially using a cross-over, prospective design, often with randomization and blinding (Davidson et al., 2021). In addition to identifying the best treatment for a person, idiographic methods of analysis can also be applied to observational data to personalize illness course models. Building on the network theory of psychopathology, an individual’s own symptom network can be modeled using ecological momentary assessments (Bringmann, 2021; Mansueto et al., 2022). Digital phenotyping can be used to generate personalized models that aim to predict a person’s deterioration in mood based on their specific mobile sensing signatures (Ren et al., 2022). Together, these approaches may ultimately help clinicians identify the optimal treatment targets (e.g., the key node of a symptom network that perpetuates depression), the most effective treatments for a person, as well as the windows of opportunities for delivering the interventions “just-in-time” (i.e., as identified by personalized predictive models that anticipate a person’s deterioration) (Nahum-Shani et al., 2018).

Addressing these challenges depends on ensuring that the right contextual variables are considered when inferring from general knowledge about how the brain works to predict individuals’ illness course and treatment response. This, in turn, depends on including adequate variation in study samples so that key contextual variables can be identified.

In addition to these challenges, which affect the potential translation of many kinds of research into clinical practice, there are several deeper issues related to how precision psychiatry proposes to make use of statistical methods and neuroscientific findings.

### Circularity and pseudo-explanation

Although a sizeable portion of the research in precision psychiatry is agnostic about causality (e.g., research that aims to identify groups of patients based on biomarkers or to generate data-driven predictions of mental illness course), observed correlations between mental health problems and biomarkers are sometimes taken to indicate a specific causal mechanism that can be targeted by precision therapeutics. For example, if a drug reduces depressive symptoms, it is deemed an “antidepressant” and its immediate mechanism of action is assumed to be part of the mechanism of disorder. This therapeutic fallacy is reminiscent of Moliere’s famous joke about a sedative working because it contains a “dormitive principle.” Neuroscience research makes heavy use of folk psychological categories and constructs to characterize both processes and outcomes (Anderson, 2015; Barrett, 2017; Dewhurst, 2021). To varying extent, this leads to circular “pseudo-explanations” which add nothing to the basic observation. Thus, many studies use a behavioral or diagnostic construct that is broad and heterogenous (e.g., “fear” or “depression”) to define a patient population and identify a neurobiological correlate of a symptom or syndrome (e.g., activity in a brain region or circuit assessed by functional neuroimaging or electrophysiology), which is then used to make claims about causal processes and therapeutic interventions. Generally, these studies are correlational and offer limited evidence to support causal or mechanistic explanations. Indeed, since the functions of specific brain regions and circuits are likely diverse and still under-characterized, these studies are open to many interpretations. In some cases, correlational studies are essentially redescriptions of plausible information-processing mechanisms based on an intuitive understanding of everyday functioning. Of course, neuroscientific research and modeling can lead to refinements in our everyday concepts of mental function (Genon et al., 2018; Bielczyk et al., 2019; Smith et al., 2022), but the relationship between these folk concepts and underlying brain mechanisms may be complex, context-dependent and many-to-many, defying any simple isomorphic mapping (Shulman, 2013; Passingham and Rowe, 2016).

Although correlational findings are the most common output of research in human neuroscience, there are research methods that enable causal inference. For example, animal models with knock-out genes have been used to demonstrate the role of interleukin-6 in neurodevelopment in mice (Phillips and Roth, 2019). Lesion studies allow localization of function in animals and humans in some clinical situations where neurosurgical interventions are needed (Vaidya et al., 2019). Studies of patient outcomes after a stroke have been used to posit the role of specific brain structures in mood and cognition (Gasquoine, 2014). However, the response to brain injury is not simply a loss of localized function but reflects compensatory responses at multiple levels, from brain plasticity and recruitment of alternate circuitry, to behavioral adaptations, and social-environmental accommodation—all of which may complicate the interpretation of the effects of even a localized acute injury (Nudo, 2013). Beyond the concerns raised above, studying causal mechanisms through longitudinal observational, experimental and statistical methods may reveal mechanisms that are distant from folk psychology and hence may be more challenging to integrate into explanatory models that are intelligible to patients.

## Social context, agency and looping effects

Crucially, causal explanation in neuroscience generally ignores individuals’ agency and subjectivity. Yet human behavior and illness experience are shaped by the ways we understand ourselves. The ways we interpret situations and events and our sense of control determine our ways of coping and adaptation and the level of stress and distress we experience. These processes of self-understanding and self-construal in turn affect the ways that patients and clinicians respond to neuroscientific explanations (Choudhury and Slaby, 2016). For example, Turnwald et al. (2019) found that learning one’s genetic risk changes physiology independent of actual genetic risk. Individuals were genotyped for genetic risk related to satiety, exercise capacity, and cardiovascular response to exercise but received assigned test results (high-risk or protected) randomly. The study found that simply being informed of one’s genetic risk changed individual’s perceived satiety and satiety physiology, as well as their cardiorespiratory physiology and perceived exercise endurance. In some cases, the effects of perceived risk on outcomes were greater than the effects associated with the actual genetic risk.

Similarly, receiving a diagnosis that conveys a specific prognosis can affect the course of illness and treatment response, in part through placebo or nocebo responses and broader expectancy effects (Pagnini, 2019; Colloca and Barsky, 2020; Özdemir and Endrenyi, 2021), as well as potentially leading to social stigma with consequences for self-efficacy, help-seeking, and employment status (Corrigan, 2018; Brouwers, 2020). Diagnostic constructs and explanations thus become social and cultural realities that shape individual experience in what the philosopher Ian Hacking (1995, 1999), Tsou (2007) has called “the looping effect of human kinds.” This effect of medical systems on experience is not unique to biological psychiatry, but it challenges efforts to characterize mental disorders independently of the ways that individuals make sense of their experience and others respond to it. Indeed, in the case of many psychiatric disorders, there may be processes of “bio-looping,” in which cognitive, behavioral, and interpersonal processes feedback to alter the individual’s neurobiology in addition to their social and psychological effects (Kirmayer, 2015b; Fuchs, 2020). The implication for research is that we need to study the interplay of self-understanding and interpersonal interactions with biomarkers, symptoms, and the course of illness and treatment response. Although computational modeling is already starting to advance a formal model of the descriptions of lived experience (Ramstead et al., 2022), the social dimensions of illness and their link to the causal processes that contribute to health and illness are not included in most studies (Dumas et al., 2020). Incorporating measures of patients’ self-understanding can improve the generalizability of findings and promote more effective clinical knowledge translation and intervention.

## Ethical issues with the use of AI and machine learning in precision medicine

Precision medicine aims to harness advances in AI and machine learning to analyze large data sets to address three broad goals (Bzdok and Meyer-Lindenberg, 2018): (i) to better describe and distinguish diagnostic entities or disorders (e.g., developing deep phenotypes that can guide advances in diagnostic nosology and clinical assessment); (ii) to infer underlying mechanisms; and (iii) to make clinically relevant predictions of course, differential treatment response, and outcome. Current pragmatic applications of precision medicine focus mainly on this predictive use. The idea here is not necessarily to better describe or understand the nature of mental disorders but to make the right clinical decision for each individual. Machine learning methods can be applied to large datasets to develop predictions that can then be used to characterize individuals in the clinic. However, when machine learning involves “black box” learning procedures in which the algorithm, logic, or evidential basis of decision-making remains opaque to clinicians, there may be critical questions about interpretability that pose ethical and practical problems (Watson et al., 2019; Fusar-Poli P. et al., 2022). These ethical and pragmatic problems include: (i) the lack of verification and external validation; (ii) failure to increase clinician knowledge and skill; (iii) lack of explainability to clinicians, patients, families and other knowledge users; and (iv) the potential for harm through incorporating existing or unknown biases (Char et al., 2020; Geneviève et al., 2020). Efforts are underway to address these issues by ensuring transparency in the construction of machine learning models, providing self-tracing reports that track decision structures, and testing models against diverse, real-world populations and scenarios for potential bias (Rasheed et al., 2022; Ratti and Graves, 2022).

These inter-related problems have a common basis and may admit a shared solution. In particular, we think an extended, multilevel, ecosocial systems approach can contribute to precision psychiatry by sharpening the clinical relevance and person-centeredness of research and by providing frameworks to more effectively translate findings into clinical practice.

## Restoring person, culture, and context to precision psychiatry

To address the limitations of current approaches, we suggest that the frameworks and research program of precision psychiatry need to be supplemented with additional constructs and measures that can situate it in relation to the social contexts and experiences that are of central concern in clinical care. At a minimum, this augmented ecosocial framework would include the following dimensions:

1. *Lifespan Developmental:* individual variation in developmental experiences, relationships, social interactions, and personal history, including child-rearing practices, life course events, transitions, and trajectories;
2. *Social-structural*: social structural (e.g., social class, racialized, or minoritized identity) and interactional determinants of adversity and patients’ predicaments, precarity, and resilience, including exposure to stigmatization, racism, discrimination, marginalization, and oppression as well as support, empowerment, and access to resources;
3. *Cultural-historical:* the situatedness or embedding of brain function in social, cultural, and historical systems of meaning, knowledge and practice that shape illness experience and explanation, coping, help-seeking, treatment expectations, and response;
4. *Experiential*: Individuals’ experience, self-understanding awareness of and meaning-based responses to symptoms and situations that draw from personal history, autobiographical narratives, social context, and cultural models in active negotiation with clinical services. Key experiences involve existential predicaments that may be a central focus of clinical concern (de Haan, 2020).

These dimensions of brain function and illness experience are neglected in most precision psychiatry research and receive limited attention in current clinical applications of neuroscientific knowledge. Advancing precision, however, is not only about adding missing dimensions but also connecting the dynamics of these dimensions in an integrative way. Moreover, all of these dimensions of illness experience, mechanisms of disorder, and functioning vary at the level of the individual, family, community, society, and culture. This diversity influences both the exposure to specific factors and the ways that they interact with individual biology. The assumption that neuroscientific findings are universally applicable ignores individual and population variability and contextual factors that contribute to the cause, mechanisms, and course of psychopathology. Recognizing the importance of context points to the need for a broader program of precision psychiatry research guided by an ecosocial approach to better capture human diversity and real-world contexts to inform clinical practice. Box 1 summarizes some key principles of an ecosocial approach to precision psychiatry.

**BOX 1 Key principles for advancing an ecosocial approach to precision psychiatry.**

**Person- and family-centered** Research and clinical practice should reflect the priorities and concerns of people with lived experience. The research community needs to better integrate feedback from stakeholders in the development of research priorities, and the design of experiments that explore the ecological niche of patients and families (Filipe et al., 2021). Clinicians need to develop processes for diagnostic assessment and treatment planning that include systematic attention to patient experience, self-understanding, and local lifeworlds (Kirmayer et al., 2016).

**Social-ecological** There is a need for a shift in perspective, away from a strictly brain-bound, individualistic approach to mental health and illness toward a more situated, embedded, interactive view of body, brain, and person in social context (Kirmayer, 2015b; Fuchs, 2017). In autism research, for example, this could include moving from studies of third-person social cognition to interactive second-person tasks (Dumas, 2022).

**Interdisciplinary** Since different dimensions, domains and levels may contribute to the etiology of mental disorders, the research community needs more collaboration across disciplines to combine expertise to investigate mental conditions from a multi-scale perspective and develop cross-level dynamic models that can inform clinical formulation and intervention (Dubé et al., 2022). Fostering interdisciplinary collaboration requires specific training opportunities for researchers and clinician, institutional structures, and incentives to promote bridging concepts and conceptual exchange (Kirmayer et al., 2020).

**Culturally diverse and representative** Research on small samples of homogenous, unrepresentative groups of patients using non-ecological laboratory-based experiments has led to poor transferability to real-world situations. A focus on generalizability would help the community generate results that more closely apply to real-world situations and improve translational efforts to bring the science to the stakeholders. The collection of “big data” provides an opportunity to remedy this focus provided that samples are representative and that measures capture crucial dimensions of variation allowing disaggregation to identify interactions specific to particular groups and contexts (Fusar-Poli L. et al., 2022). Analysis of cultural diversity can provide a powerful way to identify novel social determinants of health and mechanisms of pathology and recovery (Seligman et al., 2016).

## Toward a person-centered ecosocial neuroscience for precision psychiatry

We have argued that a multilevel approach that goes beyond the individual body, to include psychological, social and cultural contexts is needed both for research and clinical applications of precision psychiatry. The RDoC scheme recognized the importance of developmental processes and the broader temporal dimension of illness trajectories but did not elaborate on this in its initial versions. More recently, efforts have been made to augment RDoC with an explicit developmental framework that emphasizes crucial developmental questions (cf. Pacheco et al., 2022). Theories of developmental psychopathology increasingly focus on *Gene* × *Environment* and *Gene* × *Person* × *Environment* interactions (Caspi and Moffitt, 2006; Belsky et al., 2020; Zhang and Belsky, 2022), in which individual genetic and environmental factors shape the risk for psychopathology according to their combined effects at critical periods of development (Paquin et al., 2021). The ways that these factors are “combined” in predictive and explanatory models involve dynamic interactions at multiple levels. Our understanding of developmental processes has important implications for the ways we conceptualize and measure environmental and social contexts (McLaughlin et al., 2021).

Similarly, there have been recent efforts to integrate social contexts and determinants of mental health into the RDoC Framework (e.g., Carter et al., 2021; Doom et al., 2021; King et al., 2021). Attention to social-cultural contexts would add further columns to the RDoC scheme to capture salient processes at the levels of cognition, interpersonal interaction, and discourse, as well as rows representing dynamic processes that may occur as part of interactions in couples, families, or communities. In some cases, an ecosocial approach to precision psychiatry may situate dysfunction primarily in the interpersonal domain. For example, Joiner et al. (1999) have argued for the importance of interpersonal context to offer an adequate account of the causes and mechanisms of depression. However, these levels are not just independent dimensions layered one on top of the other but part of a system that gives rise to new dynamics that in turn reflect the interactions of processes within and across levels (e.g., epigenetics × childrearing practices × family environment × neighborhood; Dubé et al., 2022; Mrug et al., 2022). What is needed to capture this complexity are conceptual approaches to cross-level integration that include the role of social context, networks of relationships, and self-understanding.

In recent decades, a step toward integration has been achieved through the incorporation of models of cognitive processes into neurobiological research on the premise that these served to link biological vulnerability, social environment, and individual symptom expression (Garety et al., 2007). More recent frameworks, such as the predictive processing or active inference approach developed in computational psychiatry research, offer avenues for deeper integration across neural and sociocultural levels of description (Ramstead et al., 2016; Veissière et al., 2020; Constant et al., 2022). However, to date, these have not been elaborated in an integrative framework that includes individual phenomenology, self-understanding, and social context. We think this is essential to achieving useful precision in psychiatric practice.

Current work in 4E cognitive science suggests ways to approach this integration (Kirmayer, 2015b; Nielsen and Ward, 2018; Bolton and Gillett, 2019; de Haan, 2020). The 4E approach examines how cognitive processes depend on bodily experiences that provide a scaffolding for more elaborate abstract thought. Crucially, the notion of cognition in 4E cognitive science encompasses affective, perceptual, attentional, imaginal, and interpersonal processes. The process of *embodied* cognition involves active engagement with the environment, so that neurocognitive function is *embedded* in, *enacted* through, and *extended* into the social world. The 4E cognitive approach begins with insights from phenomenology and links them to empirical work on development and everyday cognitive functioning. Many of the theoretical claims of 4E cognitive science can be operationalized in terms of current models of active inference in computational psychiatry (Badcock et al., 2019; Hipólito and van Es, 2022). This allows us to build models of cognitive function and adaptation that include both the brain and the social world, through interactions with other people and institutions that present cultural affordances (Ramstead et al., 2016; Kirmayer and Ramstead, 2017; Veissière et al., 2020; Tison and Poirier, 2021; Constant et al., 2022).

The 4E perspective can be further elaborated by incorporating insights from social epidemiology, developmental psychology, family systems theory, and psychological anthropology to characterize the ways in which human functioning depends on social networks, niches, and interactions with others. The resultant ecosocial approach considers the brain as situated in social contexts and actively engaged in adaptation through cooperation with others by mobilizing cultural affordances. This ecosocial view gives a central place to individual agency and subjectivity as the site of personal values and a crucial target of interventions but also as an intrinsic part of causal processes. The phenomenology of illness experience is central to what drives people to seek help and to their specific clinical concerns. Modes of self-understanding, experiences and interpretations of symptoms and interactions with others through bodily and narrative forms of communication can all contribute to the emergence and evolution of mental disorders. By placing the individual as a self-reflective agent at the center of our models of mental disorders, this approach is consistent with the person-centered integrative diagnostic framework (Mezzich et al., 2010).

The person-centered approach is concerned with understanding the patient as a person in a lifeworld. Mechanisms of disorders—whether characterized in terms of the subpersonal mechanisms of neurobiology, cognitive, or interpersonal interactions—are brought together with understanding the predisposing and protective factors that modulate the course of illness. This information is integrated into a diagnostic formulation that is ecological in that it considers the dynamic properties of the multilevel system in which the individual is embedded. Neurobiology is not separate from this ecology but emerges from it and is shaped by it. Interventions at the neurobiological, cognitive, or social level affect each other. The choice of levels and locus of intervention will then be driven by pragmatic considerations of resources and feasibility as well as patients’ values and preferences.

Developing a situated view of the brain as part of larger ecosocial systems is essential to ensuring that our models of mental disorders and interventions are fit for the task of delivering person-centered care. This will allow us to study and treat mental disorders with the attention of their personal, social, and cultural context. This requires systems thinking, in which the process of illness evolution, adaptation, and recovery are understood as constituted by networks of interaction within and beyond the brain. These networks have developmental and social histories that shape experience as well as environmental contingencies, and they also give individuals narrative frameworks within which to understand and reflect on their symptoms and illness experience. This self-understanding and collective social-cultural framing then loops back to influence how individuals adapt to their condition.

Recognizing the role of phenomenology, self-understanding, and cultural meaning in illness experience opens the door to a precision psychiatry that will engage with social variation, intersectionality, and cultural diversity. From the ecosocial perspective, these dimensions of experience are not supplements to basic neurobiological mechanisms, added on after the fact to tailor interventions, but instead are seen as constitutive of illness experience and the mechanisms of disorder and recovery. Hence, they must be studied in concert with neurobiological mechanisms.

An ecological view adds explicit attention to the contexts in which we live, through examining niche construction and the dynamics of larger networks of social systems in which local worlds are embedded. A person-centered approach adds attention to subjectivity, agency, values, relationality, and the looping effects that result from our capacities for self-reflection.

Capturing the missing dimensions of personhood will not only ensure that research takes into account crucial health determinants but, by allowing statistical control for individual variation, aid in the process of identifying mechanisms of pathology and recovery at biological as well as psychological and social levels. To do this, of course, we need real interdisciplinarity in the design, interpretation, and translation of research. This needs to go beyond the collaborations between biology and AI currently framed as *convergence science* (National Research Council, 2014), to include fields focused on the systematic characterization of lived experience and social context (Eyre et al., 2017; Kirmayer et al., 2020; Eyre et al., 2021; Dubé et al., 2022).

Achieving the necessary interdisciplinarity must begin at the pedagogical level. The integration of ecosocial approaches in precision psychiatry requires a new generation of scientists and clinicians trained in very different disciplines such as computational medicine and social determinants of health. This will require multi-disciplinary curricula with project-oriented programs that allow students to be confronted early on with challenges at both technical, clinical, and human levels. The discipline-bound orientation of many academic institutions needs to be complemented with truly interdisciplinary settings where researchers, clinicians, and stakeholders can develop bridging theories and corresponding methods to address the core dimensions of personhood in health and illness. The reward structures of academia and the review processes of both grant funding and scientific publication also need rethinking to promote interdisciplinarity (Kirmayer et al., 2020).

## Conclusion

Precision psychiatry looks to neuroscience to lay bare the underlying mechanisms of mental disorders and, more immediately, to allow us to tailor treatment interventions with greater precision through the measurement of biological parameters. However, characterizing individuals only in terms of biological variables may yield very limited precision, with individuals lumped together in categories that may be predictive of a particular facet of treatment response (e.g., drug side-effects) but that ignore many other aspects of individual variation that interact with neurobiology in fundamental ways. The dynamic interaction of neurobiology and social context is central to predicting illness course and the response to both pharmacological and psychosocial interventions.

In biomedical research, neuroscientific models of psychiatric disorders are judged in terms of their fit with data, their explanatory power, and their ability to generate predictions and hypotheses for further studies. To be useful in clinical contexts, models and explanations derived from precision psychiatry research need to be examined in terms of the kinds of clinical thinking and case formulation they enable, and the ways that they function when conveyed to patients.

Diagnostic categories or syndromes have practical utility for capturing similarities between individual presentations, an essential process for population-level inference in research and clinical practice. However, a person’s characteristics, experiences, and context can never be fully “measured,” and thus one needs to make pragmatic decisions to narrow the focus in research or clinical assessment to salient features of the person’s health condition. Fortunately, there are alternatives to diagnostic categorization for addressing these challenges. First, clinical staging can be used to describe a person’s mental health problems longitudinally (Shah et al., 2020). This approach draws on the observation that symptom combinations tend to cut across diagnostic categories and fluidly evolve over time. Clinical staging emphasizes the relevance of illness severity and its longitudinal course for guiding research, service organization, and clinical care. A second, complementary approach aims to describe psychopathology dimensionally, which can confer greater reliability and precision than traditional diagnostic systems, and which may help identify intervention strategies (Kotov et al., 2020). Current work on symptom network theory provides a way to explore such dimensional models by examining the associations between symptoms that may give rise to clinical syndromes through their interactions (Borsboom, 2017: Bringmann et al., 2022). This approach is readily extended to incorporate social and environmental factors as risk and protective factors or as part of pathogenic network dynamics (Lunansky et al., 2021). Third, measurement methods and foci of clinical inquiry should be designed and selected in concert with patients and communities (Fried et al., 2022). Through collaborative investigations into the longitudinal and dimensional aspects of mental illness, researchers, clinicians and patients may overcome the limitations of diagnostic systems and come closer to the ideals of person-centered, precision psychiatry.

For clinicians, a useful case formulation yields reliable predictions about course and outcome and indicates appropriate treatment strategies and specific interventions. For patients, diagnostic labeling and formulation serve additional functions—providing a workable explanation that answers basic questions about the nature of their suffering and appropriate guidance, sources, and forms of help. Of course, patients are not simply passive recipients of explanations in this process but actively seek out information and ways of making sense of their problems. Hence, neuroscientific explanations are used by patients to understand and cope with their symptoms and suffering, and to communicate it to others in ways that may have both helpful and damaging consequences. This search for meaning occurs in the clinical context and beyond, in conversation with others who offer ideas, share experiences, or present examples drawn from an ecology of information that circulates through the popular media, the Internet, and wider social networks and institutions (Choudhury and Slaby, 2016).

To be of maximum clinical utility and avoid over-generalization, then, the neuroscientifically based explanations and interventions sought by precision psychiatry must be situated in a larger ecosocial, systemic view of symptom networks, interpersonal interactions, and adaptations. More integrative multi-level system approaches can begin to realize the original promise of the biopsychosocial approach by showing how neurobiological models can be integrated with close attention to the social-cultural contexts that give rise to psychiatric disorders. This integrative approach can bridge the precision of mechanistic explanation with the person-centeredness of phenomenology in research and practice.

Precision in psychiatric diagnosis and treatment will depend on characterizing how these multilevel processes interact in specific individual developmental trajectories and social contexts. A reductive neuroscience that does not consider cognitive and social processes cannot bridge these gaps since it does not sufficiently engage with precisely the levels of processes where problems and solutions may reside. Indeed, framing personalized medicine and psychiatry mainly in terms of greater precision in assessing individual biological variation leads to modes of clinical care that tend to ignore individuals’ experience as well as their sociocultural and historical context. The elision of the person’s experience and self-understanding leads to an inadequate characterization of the nature of pathology and the resources for healing and recovery. Efforts to achieve “precision” in psychiatric practice should never be at the cost of the clinical relationship. The use of computational tools should facilitate the process of differential diagnosis and allow the collection of clinically relevant information that may not be readily observable by a clinician, but ultimately this is aimed at supporting the interpersonal process of clinical care. This requires developing a thoroughgoing person-centered and ecosocial approach that considers the person in social context in a lifespan developmental framework. Neuroscience can then be incorporated into a context-sensitive, systemic view that includes patients’ experience not only as crucial data about their health needs, priorities, and concerns, but as the medium through which clinical communication, collaboration, and intervention must occur.

## Data availability statement

The original contributions presented in the study are included in the article/supplementary material, further inquiries can be directed to the corresponding author.

## Author contributions

AG-C and LK contributed equally to the conception of the manuscript. AG-C wrote the first draft of the manuscript. LK wrote sections of the manuscript. AG-C, LK, VP, and GD reviewed the manuscript critically for intellectual content. All authors contributed to manuscript revision, read and approved the submitted version.

## Footnotes

1
https://www.nimh.nih.gov/research/research-funded-by-nimh/rdoc
